# The Adhesion GPCR ADGRL2/LPHN2 Can Protect Against Cellular and Organismal Dysfunction

**DOI:** 10.3390/cells13221826

**Published:** 2024-11-05

**Authors:** Philipp Jakobs, Anne Rafflenbeul, Willem Berend Post, Niloofar Ale-Agha, Victoria Elisabeth Groß, Stephanie Pick, Sascha Dolata, Fiona F. Cox, Florian von Ameln, Olaf Eckermann, Joachim Altschmied, Simone Prömel, Judith Haendeler

**Affiliations:** 1 Cardiovascular Degeneration, Haendeler Group, Clinical Chemistry and Laboratory Diagnostics, Medical Faculty, University Hospital and Heinrich Heine University Düsseldorf, 40225 Düsseldorf, Germany; philipp.jakobs@hhu.de (P.J.); niale001@hhu.de (N.A.-A.); olaf.eckermann@hhu.de (O.E.); 2 Cardiovascular Degeneration, Altschmied Group, Clinical Chemistry and Laboratory Diagnostics, Medical Faculty, University Hospital and Heinrich Heine University Düsseldorf, 40225 Düsseldorf, Germany; anne.rafflenbeul@hhu.de (A.R.); fiona.cox@hhu.de (F.F.C.); florian.ameln@hhu.de (F.v.A.); joalt001@hhu.de (J.A.); 3 Institute of Cell Biology, Department of Biology, Heinrich Heine University Düsseldorf, 40225 Düsseldorf, Germany; postw@hhu.de (W.B.P.); grossv@hhu.de (V.E.G.); stephanie.pick@hhu.de (S.P.); sascha.dolata@hhu.de (S.D.)

**Keywords:** ADGRL2/LPHN2, *Caenorhabditis elegans*, endothelial cells, eNOS, lipopolysaccharide, NRF2, *skn-1*, reactive oxygen species

## Abstract

The most common trigger of sepsis and septic shock is bacterial lipopolysaccharide (LPS). Endothelial cells are among the first to encounter LPS directly. Generally, their function is closely linked to active endothelial NO Synthase (eNOS), which is significantly reduced under septic conditions. LPS treatment of endothelial cells leads to their activation and apoptosis, resulting in loss of integrity and vascular leakage, a hallmark of septic shock. Hence, therapies that prevent endothelial leakage or restore the endothelial barrier would be invaluable for patients. Adhesion GPCRs (aGPCRs) have been largely overlooked in this context, although particularly one of them, ADGRL2/LPHN2, has been implicated in endothelial barrier function. Our study shows that overexpression of ADGRL2 protects endothelial cells from LPS-induced activation, apoptosis, and impaired migration. Mechanistically, ADGRL2 preserves eNOS activity by shifting its binding from Caveolin-1 to Heat Shock Protein 90. Furthermore, ADGRL2 enhances antioxidative responses by increasing NRF2 activity. Notably, we found that this function may be evolutionarily conserved. In the absence of *lat-2*, a homolog of ADGRL2 in *Caenorhabditis elegans*, worms show higher ROS levels and altered stress response gene expression. Additionally, *lat-2* mutants have a significantly reduced lifespan, altogether indicating a protective role of ADGRL2 against oxidative stress across species.

## 1. Introduction

Sepsis can best be described as an overshooting immune response to a microbial infection, which can lead to shock, multiple organ failure, and often death. In 2017, the World Health Organization (WHO) estimated that sepsis affects more than 48 million individuals every year, potentially leading to 11 million deaths annually [1]. The basis for the pathophysiological responses in sepsis and septic shock is multifactorial, which might serve as an explanation why—besides vasopressor agents, which have been in use for several decades—no new therapeutic approaches have been developed for sepsis treatment to date.

The most common trigger for sepsis and septic shock is bacterial lipopolysaccharide (LPS), a serologically reactive bacterial toxin. It is a component of the outer membrane of Gram-negative bacteria, which triggers a strong proinflammatory response in the human immune system, with 1 to 2 mg in the bloodstream potentially being lethal [2]. LPS can enter the bloodstream through intestinal absorption of the LPS produced by gut bacteria. Moreover, gut lesions and diets rich in lipids boost the transport across membranes into the systemic circulation [3]. Therefore, at the cellular level, endothelial cells (ECs) are, besides circulating immune cells, the first cells directly confronted with LPS.

Endothelial functionality is intrinsically tied to the availability of NO produced by endothelial NO Synthase (eNOS). Under septic conditions or during chronic inflammation, this enzymes’ activity is severely reduced [2,4]. Moreover, LPS triggers endothelial cell activation, a proinflammatory and procoagulant state of the endothelium, and ultimately apoptosis, resulting in loss of the endothelial integrity and vascular leakage, a mainstay of septic shock [2]. Hence, therapies, which could prevent endothelial cell leakage or even restore the endothelial barrier, would be of enormous value for patients and would address medical needs. However, up to now, the molecular mechanisms by which LPS leads to endothelial activation, apoptosis, and leakage are far from understood.

One class of molecules, which role in endothelial functionality has been underestimated to date, is Adhesion G protein-coupled receptors (aGPCRs). They combine cellular adhesion with intracellular signaling. Many features of aGPCRs, such as their ability to mediate cell–cell interactions and adhesion, makes them interesting candidates to be involved in EC function. Intriguingly, three aGPCRs, which are expressed in ECs, have been firmly linked to their function to date: ADGRF5/GPR116, ADGRL2/LPHN2, and ADGRL4/ELTD1, with the latter two belonging to the group of Latrophilins, which also contains ADGRL1/LPHN1 and ADGRL3/LPHN3 [5].

With respect to barrier function, only ADGRL2 seems to play a role in ECs [6,7]. It is localized at cell-to-extracellular matrix contacts and promotes tight junction assembly. Vascular ECs of ADGRL2-deficient zebrafish embryos are abnormally stretched and, thus, hyperpermeable, such that cells transported by the blood stream extravasate more easily into the surrounding tissues [6]. Further, ADGRL2 regulates flow-induced changes and barrier functions of ECs [7]. These studies suggest essential functions of this aGPCR in the regulation of endothelial integrity and raise the question of whether it also plays a role in LPS-induced endothelial dysfunction.

In the present study, we found protective functions of the Latrophilin ADGRL2 in human ECs, and those are, in part, evolutionarily conserved as *C. elegans* lacking the Latrophilin homolog LAT-2 and have reduced functionality.

## 2. Materials and Methods

### 2.1. Cell Culture

Primary human ECs were obtained from LONZA and cultured in endothelial basal medium supplemented with 50 ng/mL amphotericin B, 1 μg/mL hydrocortisone, 50 μg/mL gentamicin, 12 μg/mL bovine brain extract, 10 ng/mL epidermal growth factor (LONZA, Cologne, Germany), and 10% fetal bovine serum until passage 3 was reached in a humidified incubator at 37 °C in an atmosphere containing 5% CO_2_.

### 2.2. Plasmids

To generate a NRF2-dependent firefly luciferase reporter vector, two copies of a 41 bp DNA fragment containing the antioxidant response element (ARE) from the murine Glutathione S-transferase alpha 1 gene promoter [8] were inserted between the *Hind III* and *Bam HI* sites of pTATA LUC+ [9] to generate p(ARE)_2_TATA-LUC. Then, this tandem ARE was placed directly upstream of the minimal promoter derived from the herpes simplex virus thymidine kinase gene. As specificity control, several point mutations, which had been shown to interfere with the activation of a minimal promoter and/or inducibility by tert-butylhydroquinone conferred by this ARE [8,10], were introduced. In addition, an invariable A in the ARE consensus derived from polymorphic AREs in the human genome [11] was replaced by a G. These mutations in the resulting plasmid p(AREmut)_2_TATA-LUC prevented the binding of NRF2 to the mutated ARE. The identity of the newly created plasmids was confirmed by restriction analysis; in addition, the regions containing the AREs were verified by DNA sequencing.

To generate a vector suitable for *ADGRL2* expression in primary human ECs, the SV40 promoter in the pcDps backbone of the previously described expression vector [12] was replaced with the human cytomegalovirus (CMV) immediate early promoter and enhancer. Therefore, a 1.4 kbp *Sca* I/*Eco* RV fragment from pcDNA3.1/myc-His(-)C (Thermo Fisher Scientific, Dreieich, Germany) containing part of the ampicillin resistance gene and the CMV promoter/enhancer was isolated and inserted into the backbone of the pcDps-based expression vector cut with *Sca* I and *Stu* I using the Blunt/TA Ligase Master Mix (New England Biolabs, Frankfurt, Germany). The identity of the newly created expression vector was verified by restriction analysis. Complete plasmid sequences are available upon request.

### 2.3. Transient Transfection of ECs

Transient transfections of ECs were performed with Effectene (Qiagen, Hilden, Germany). In detail, cells were transfected on 6 cm culture dishes with 2.5 µg plasmid DNA, 20 µL Enhancer, and 25 µL Effectene in 150 µL buffer; subsequently, 1 mL cell culture medium was added. For transfections of luciferase reporter plasmids, 0.25 µg of these were used and made up to 2.5 µg with either an empty vector or the *ADGRL2* expression vector. For transfections in 6-well culture plates, 1 µg plasmid DNA, 8 µL Enhancer, and 10 µL Effectene in 100 µL buffer were used; subsequently, 300 µL medium were added.

### 2.4. Generation of Sequence Logos

The ARE sequence logo was generated using WebLogo 3 (https://weblogo.threeplusone.com/, accessed on 10 October 2024 [13]) using the matrix profile for the human NRF2-binding site (MA0150.1) from JASPAR 2024 [14].

### 2.5. Migration Assay

The migration of ECs was quantitated by the scratch wound assay; therefore, the cells were cultivated on 6 cm dishes. Before a wound was set by scraping off a section of the monolayer with a sterile disposable rubber policeman, the bottom of the dishes was labeled with a trace line indicating the wound position. After setting the injury, detached cells were removed by a gentle washing step with culture medium, and 20 h later, the cells were fixed with 4% (*w*/*v*) formaldehyde for 15 min at room temperature and stained with 500 ng/mL 4′,6-diamidino-2-phenylindole (DAPI) (Carl Roth, Karlsruhe, Germany) in PBS, and microscopic pictures were taken using a Zeiss Axio Observer 7. The cells, which had migrated into the wound from the trace line, were automatically counted using the particle analysis feature of ImageJ [15] after watershed separation of overlapping nuclei.

### 2.6. Immunoblotting

Cells were harvested by detaching them from the culture surface with a rubber policeman followed by centrifugation at 800× *g*. After washing the cells twice with ice cold PBS, the supernatant was completely removed. The pelleted cells were resuspended in radioimmunoprecipitation assay (RIPA) buffer (50 mM Tris-HCl, pH 8.0, 150 mM NaCl, 1% (*v*/*v*) IGEPAL-CA630, 0.1% (*w*/*v*) SDS, and 0.5% (*w*/*v*) Na-deoxycholate) supplemented with 1/100 volume of a protease inhibitor cocktail and phosphatase inhibitor cocktail (Bimake, Munich, Germany). The cell suspension was placed on ice for 30 min for lysis, before the lysates were centrifuged at 18,000× *g* and 4 °C for 15 min. The supernatants containing the cellular proteins were transferred to a fresh tube. Proteins were separated by standard sodium-dodecyl-sulfate polyacrylamide gel electrophoresis and blotted onto polyvinylidene difluoride membranes by wet transfer. The membranes were blocked with 5% (*w*/*v*) milk powder in TBS (200 mM Tris-HCl, pH 8.0, 300 mM NaCl, and 100 mM KCl) with 0.1% (*v*/*v*) Tween-20 (TBS-T) for 1 h at room temperature before they were incubated with primary antibodies overnight at 4 °C. The following day, membranes were washed three times with TBS-T and incubated with horseradish peroxidase-coupled secondary antibodies for 2 h at room temperature. Detection was performed using ECL substrate (Cytiva, Marlborough, MA, USA) and X-ray films. Semi-quantitative analyses were performed on scanned X-ray films using ImageJ 2.9.0. [15]. All antibodies used for immunoblotting are listed in Supplementary Table S1.

### 2.7. Immunostaining

ECs were seeded on acidified glass slides in 6-well culture plates and transfected as described under Section 2.3, and 5 h after transfection, the cells were treated as indicated, and 20 h later, they were fixed with 4% (*w*/*v*) formaldehyde for 15 min at room temperature. This was followed by a blocking/permeabilization step with 3% (*v*/*v*) normal goat serum (Sigma-Aldrich, Deisenhofen, Germany) diluted in PBS containing 0.3% (*v*/*v*) Triton X-100 for 15 min at room temperature. Then, cells were incubated with primary antibodies overnight at 4 °C and subsequently washed three times with PBS. After that, they were incubated with Alexa Fluor-coupled secondary antibodies for 1 h at room temperature. Nuclei were counterstained with DAPI (500 ng/mL) (Carl Roth, Karlsruhe, Germany) in PBS for 5 min at room temperature. Finally, cells were mounted with ProLong Diamond Antifade Mountant (Thermo Fisher Scientific, Dreieich, Germany). Images were taken using Zeiss Axio Observer 7 (magnification 400× or 200×), and pixel intensities were measured with ImageJ 2.9.0. [15]. All antibodies used for immunostaining are listed in Supplementary Table S2.

### 2.8. Luciferase Assay

The day after transfection, the cells were lysed with Reporter Lysis Buffer (Promega, Mannheim, Germany) according to the manufacturer’s instructions. Identical amounts of cellular protein were made up to 30 μL with Reporter Lysis Buffer, and luciferase activity was measured by the automatic injection of 100 μL Luciferase Assay Reagent (Promega, Mannheim, Germany) using a LB 960 CENTRO XS3 Luminometer (Berthold, Bad Wildbad, Germany).

### 2.9. Proximity Ligation Assay

Up to the incubation with the primary antibodies, all steps were identical to the procedures described under Section 2.7. *Immunostaining*. After overnight incubation at 4 °C with the primary antibodies, cells were treated with the components of the Duolink In Situ detection kit (Sigma-Aldrich, Deisenhofen, Germany) according to the manufacturer’s instructions. The actin cytoskeleton was stained with Phalloidin CF488A (1:70) (Biotium/Biomol, Hamburg, Germany) for 20 min at room temperature; nuclei were stained with DAPI (500 ng/mL) (Carl Roth, Karlsruhe, Germany) in PBS for 5 min at room temperature. The primary antibodies were the same as for immunostaining and are listed in Supplementary Table S2.

### 2.10. C. elegans Maintenance and Strains

*C. elegans* were maintained at 22 °C on nematode growth medium (NGM) agar plates seeded with *E. coli OP50* according to standard methods [16]. Experiments were performed at 22 °C unless stated otherwise. Wild-type worms were *C. elegans* var. Bristol strain N2 [16], and *lat-2(knu505(8932 bp deletion)) II* was generated by NemaMetrix Inc. (Eugene, OR, USA) using CRISPR/Cas9 genome editing deleting the entire *lat-2* coding sequence.

### 2.11. Lifespan Measurements

Synchronized L4 hermaphrodites were transferred to fresh plates daily during the reproductive period and every third day thereafter. Nematode viability was assessed every 2–3 days. An individual was considered dead if there was no movement or response to touch. Those that died from rupture, internal bagging, or were missing were censored from the data.

### 2.12. Brood Size Assays

L4 hermaphrodites were placed separately on NGM agar plates seeded with *E. coli OP50* and allowed to lay eggs. The individuals were transferred to a fresh plate every 24 h until egg-laying ceased, and eggs were counted. Plates were incubated at 22 °C. To determine survival to adulthood, the number of adult animals was scored 48 h after the mother was removed.

### 2.13. ROS Quantification in C. elegans

To study ROS levels in nematodes under normal, as well as stress, conditions, ROS were quantified using H_2_DCF-DA (Thermo Fisher Scientific, Dreieich, Germany), which readily permeates cell membranes. Bleach-synchronized L4 + 1-day-old nematodes were washed off plates, collected, washed three times with M9 to remove bacteria, and exposed to either 100 µM juglone (in ethanol, prepared fresh before every assay) or vehicle-only (0.9% ethanol) conditions for 1 h in PBS-Tween20 (0.1%) (PBS-T). Worms were washed three times with PBS-T and freeze-cracked once in liquid nitrogen. Sonication was applied for 1 min to disrupt cellular membranes followed by centrifugation at 21,000× *g* for 15 min. Protein amounts were determined with a Bradford assay using RotiQuant (Carl Roth, Karlsruhe, Germany). Subsequently, 50 µL of supernatant was added to a black, clear-bottom 96-well plate, and 50 µL 100 µM H_2_DCF-DA was added. Samples were mixed and incubated for 30 min, followed by a 2 h kinetic measurement of the fluorescence levels (excitation: 485 ± 25 nm, emission: 535 ± 20 nm). The ROS levels were quantified by subtracting end measurement values from the starting values and normalizing for nematode quantity based on the protein concentrations determined by the Bradford assay.

### 2.14. Isolation of Total RNA

Total cellular RNA from ECs was isolated as previously described [17]. In detail, TRIzol (Thermo Fisher Scientific, Dreieich, Germany) was used to extract total RNA from ECs according to the manufacturer’s instructions. Further purification of RNA was achieved by using the RNeasy Mini kit (Qiagen, Hilden, Germany), and concentrations were measured using a NanoDrop^TM^ 2000c (Thermo Fisher Scientific, Dreieich, Germany). RNA integrity and purity were analyzed by agarose gel electrophoresis. Nematode RNA was isolated by washing young adult nematodes off NGM plates and washing thrice with M9 buffer. The worms were subsequently resuspended in 200 µL TRI Reagent (Thermo Fisher Scientific, Dreieich, Germany) and snap-frozen in liquid nitrogen, followed by freeze–thawing three times. RNA was isolated using the Direct-zol RNA MiniPrep kit (Zymo Research, Freiburg, Germany) according to the manufacturer’s instructions and checked for integrity by agarose gel electrophoresis.

### 2.15. cDNA Synthesis

RNA from ECs was reverse-transcribed using the QuantiTect Reverse Transcription kit (Qiagen, Hilden, Germany) according to the manufacturer’s instructions. cDNA synthesis from *C. elegans* samples was performed using the Luna Script RT Master Mix with Oligo d(T)23 VN primers (both New England Biolabs, Frankfurt, Germany). A total of 500 ng of RNA was transcribed into cDNA following the manufacturer’s instructions.

### 2.16. Polymerase Chain Reaction (PCR)

The transcript levels in ECs were determined by semi-quantitative real-time PCR with cDNA as template and the primaQUANT 2× qPCR-SYBR-Green-Master Mix (Steinbrenner, Wiesenbach, Germany); the transcript for the ribosomal protein L32 (*RPL32*) was used for normalization. Thermal cycling was performed in a Rotor-Gene Q instrument (Qiagen, Hilden, Germany). Relative expression was calculated by the ΔC_t_ method [18]. Homogeneity of the reaction products was analyzed by melting point analysis, and their size was confirmed by standard agarose gel electrophoresis. Transcript levels of target genes from nematode samples were determined by qPCR using the Luna Universal qPCR Master Mix (New England Biolabs, Frankfurt, Germany) according to the manufacturer’s instructions. cDNA from each sample was diluted 1:10 with nuclease-free water, and 2 µL of this diluted cDNA was used in each qPCR reaction. To normalize the gene expression levels, the housekeeping genes *act-1*, *cdc-42*, *pmp-3*, *eif-3.c*, and *tba-1* were used. C_t_ values of these housekeeping genes were averaged to provide a robust normalization factor. Relative quantification of target gene expression was carried out using the ΔΔC_t_ method, as described by [19]. For the primer sequences, see Supplementary Table S3.

### 2.17. Statistics

The number of experiments (n) represents independent biological replicates. The data presented are the mean ± SEM. Normal distribution was confirmed by the Shapiro–Wilk test, and homogeneity of the variances (from means) was verified by Levene’s test. Since all data sets represented normal distribution, the multiple comparisons were performed using one-way ANOVA with post hoc Tukey LSD test or two-way ANOVA with post hoc Bonferroni test (for *C. elegans* ROS assays) or Student’s *t*-test (for *C. elegans* assays). *C. elegans* lifespan analyses were displayed as Kaplan–Meier curves and a Cox–Mantel test was performed to test for significance. Details are given in the respective figure legends.

## 3. Results

### 3.1. ADGRL2 Expression Is Reduced in ECs in Response to LPS

**Figure 1:**
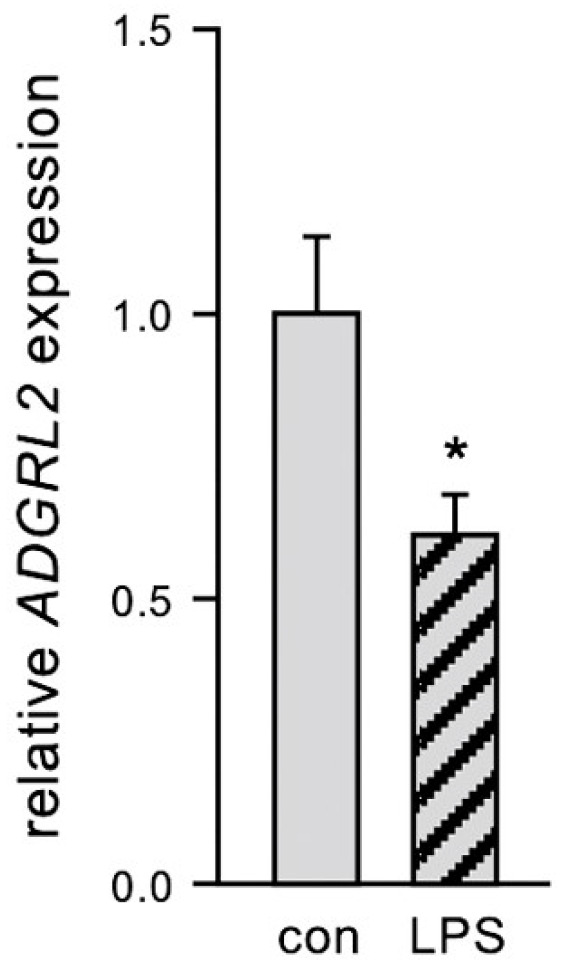
LPS downregulates *ADGRL2* expression in primary human ECs. ECs were treated for 20 h with 150 ng/mL active LPS (LPS) or detoxified LPS as the control (con). Expression of *ADGRL2* was determined by semi-quantitative real-time PCR. *RPL32* was used for normalization (data are the mean ± SEM, n = 4, * *p* < 0.05 vs. con, unpaired, two-sided Student’s *t*-test).

To investigate the effects of LPS on *ADGRL2* expression in ECs, we first treated primary human ECs with 150 ng/mL active LPS or detoxified LPS as the control. As shown in Figure 1, *ADGRL2* expression was significantly reduced in response to LPS.

We had previously published that LPS induces activation and apoptosis [17] and reduces the migratory capacity of ECs [20]—all of them hallmarks of endothelial dysfunction. Based on the downregulation of *ADGRL2*, we hypothesized that its overexpression could counteract LPS-induced dysfunctionality of ECs.

### 3.2. ADGRL2 Overexpression Inhibits LPS-Induced EC Activation, Apoptosis, and Preserves Migratory Capacity

**Figure 2:**
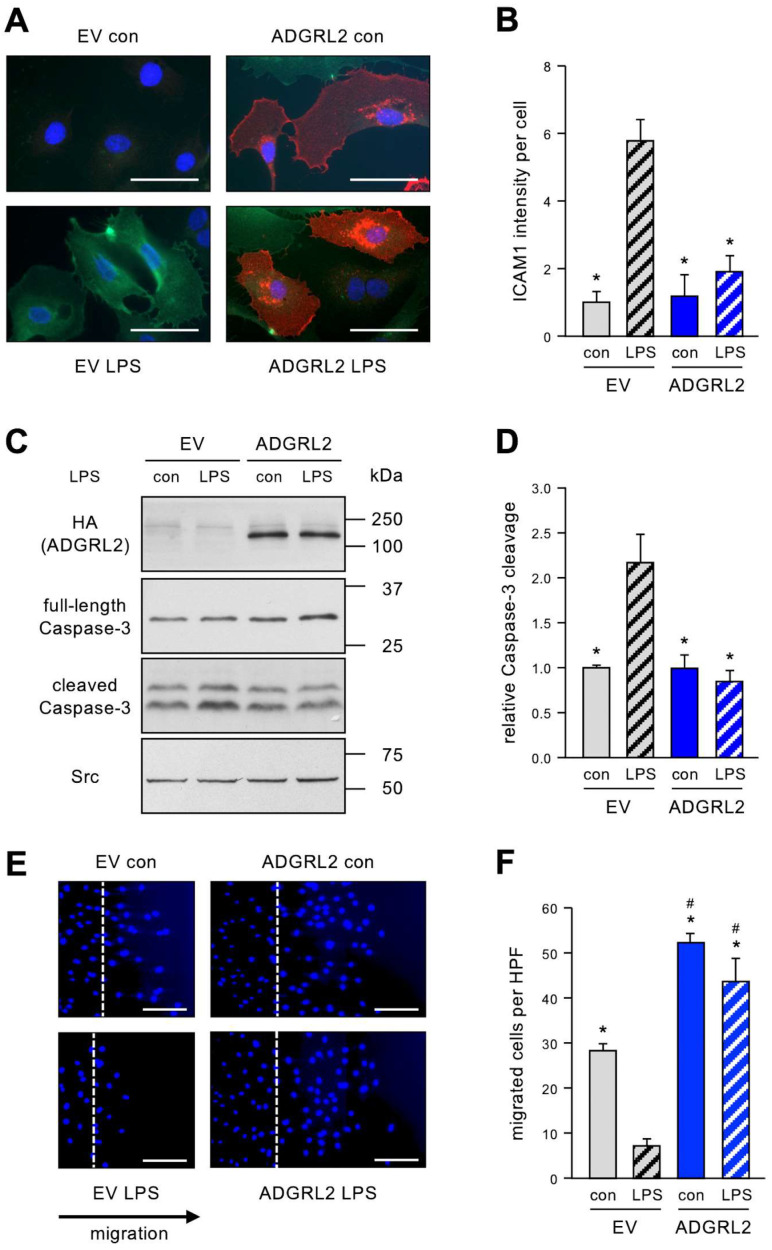
ADGRL2 prevents LPS-induced activation and apoptosis of ECs and enhances the migratory capacity. ECs were transfected with an expression vector for ADGRL2 or a corresponding empty vector (EV). After transfection, the cells were treated for 20 h with 150 ng/mL active LPS (LPS) or detoxified LPS as the control (con). (**A**,**B**) Cells were fixed and stained for ICAM1 (green) and ADGRL2 (red), and nuclei were counterstained with DAPI. (**A**) Representative immunofluorescence staining (scale bars = 50 μm). (**B**) Semi-quantitative analysis of ICAM1 levels per cell (data are the mean ± SEM, n = 4, * *p* < 0.05 vs. EV/LPS, one-way ANOVA with post hoc Tukey LSD test). (**C**,**D**) HA-tagged ADGRL2 (HA(ADGRL2)), uncleaved (full-length Caspase-3), and cleaved Caspase-3 were detected by immunoblot, and Src served as the loading control. (**C**) Representative immunoblots. (**D**) Semi-quantitative analysis of relative amounts of cleaved Caspase-3 (data are the mean ± SEM, n = 5, * *p* < 0.05 vs. EV/LPS, one-way ANOVA with post hoc Tukey LSD test). (**E**,**F**) The migratory capacity was determined by scratch wound assays. Cell nuclei were stained with DAPI. (**E**) Representative DAPI staining. Wounds were set at the dashed lines; right of the lines are the cells that had migrated into them (scale bars = 100 µm). (**F**) Semi-quantitative analysis of migrated cells per high-power field (HPF) (data are the mean ± SEM, n = 5–6, * *p* < 0.05 vs. EV/LPS, # *p* < 0.05 vs. EV/con, one-way ANOVA with post hoc Tukey LSD test).

To study the impact of ADGRL2 on endothelial cell functions affected by LPS, we generated an expression vector, which contained HA (N-terminus) and FLAG (C-terminus)-epitope tags allowing the identification of the overexpressed protein. We then investigated the effects of HA-ADGRL2-FLAG on EC activation, apoptosis, and migration. Therefore, HA-ADGRL2-FLAG was expressed in ECs, and ECs were treated with 150 ng/mL LPS for 20 h. For EC activation, ICAM1 (Intercellular Adhesion Molecule 1), a typical marker, was detected. As expected LPS induced an elevation in ICAM1 levels in empty vector (EV) transfected cells. This upregulation was completely inhibited in cells in which HA-ADGRL2-FLAG is overexpressed (Figure 2A,B).

Next, we determined the effect of HA-ADGRL2-FLAG on apoptosis and used cleavage of the effector Caspase-3 as the marker. As for ICAM1, LPS induced Caspase-3 cleavage in EV transfected cells, whereas overexpression of HA-ADGRL2-FLAG blunted apoptosis induction by LPS (Figure 2C,D).

Finally, we measured the migratory capacity of ECs, and 5 h after transfection, a wound was set into the EC layer and cells were treated with LPS for 20 h. As shown in Figure 2, HA-ADGRL2-FLAG not only inhibited LPS-induced impairment in the migratory capacity but also significantly improved migration when compared to EV transfected cells (Figure 2E,F).

### 3.3. ADGRL2 Overexpression Counteracts the LPS-Induced Reduction in Endothelial NO Synthase Activity

**Figure 3:**
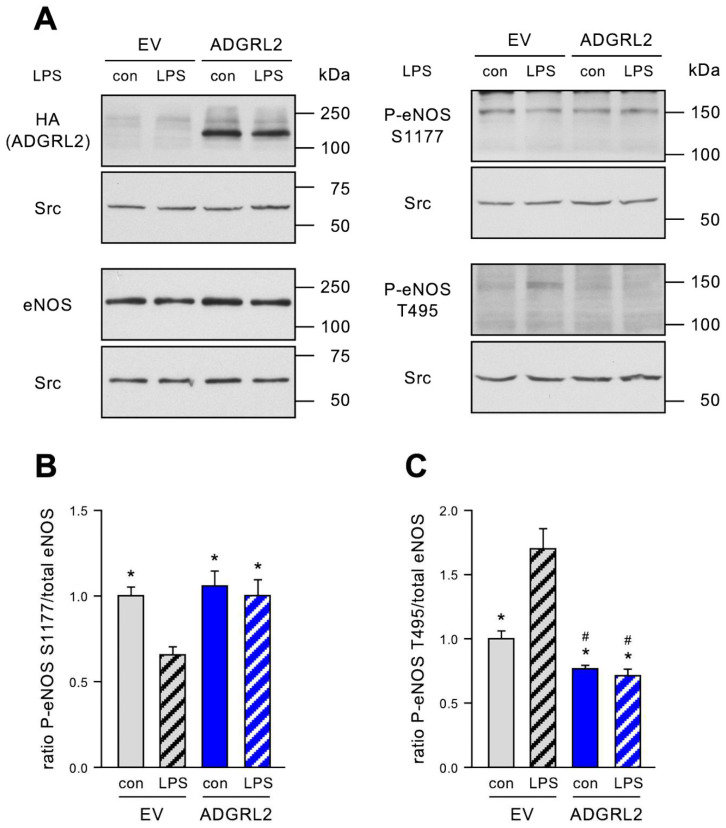
ADGRL2 increases eNOS activity. ECs were transfected with an expression vector for *ADGRL2* or a corresponding empty vector (EV). After transfection, the cells were treated for 20 h with 150 ng/mL active LPS (LPS) or detoxified LPS as the control (con). HA-tagged ADGRL2 (HA (ADGRL2)), total eNOS (eNOS), and eNOS phosphorylated at serine 1177 (P-eNOS S1177) or at threonine 495 (P-eNOS T495), respectively, were detected by immunoblot; in all cases, Src served as the loading control. (**A**) Representative immunoblots; each blot is shown with its respective loading control. (**B**) Semi-quantitative analysis of the ratio of eNOS phosphorylated at serine 1177 to the total eNOS (data are the mean ± SEM, n = 8, * *p* < 0.05 vs. EV/LPS, one-way ANOVA with post hoc Tukey LSD test). (**C**) Semi-quantitative analysis of the ratio of eNOS phosphorylated at threonine 495 to total eNOS (data are the mean ± SEM, n = 5, * *p* < 0.05 vs. EV/LPS, # *p* < 0.05 vs. EV/con, one-way ANOVA with post hoc Tukey LSD test).

Activation and apoptosis induction, as well as the impaired migratory capacity of ECs, are all linked to reduced NO bioavailability. Thus, we next investigated the phosphorylation states of the eNOS as a surrogate marker for its activity. Phosphorylation of serine 1177 (S1177) activates the enzyme, whereas phosphorylation of threonine 495 (T495) inactivates it [21]. HA-ADGRL2-FLAG was expressed in ECs, and cells were again treated with 150 ng/mL LPS. As expected, LPS reduced the phosphorylation of eNOS S1177 and increased the phosphorylation of T495 (Figure 3), demonstrating a reduction in active eNOS and, thereby, NO bioavailability. HA-ADGRL2-FLAG completely inhibited LPS-induced dephosphorylation of eNOS S1177, as well as phosphorylation of eNOS T495 (Figure 3), indicating the preservation of enzyme activity. Interestingly, the phosphorylation of eNOS T495 was also reduced when compared to EV transfected cells treated with detoxified LPS (Figure 3C). Thus, one could assume that overexpression of ADGRL2 already under basal conditions increases NO bioavailability.

For a deeper understanding of the mechanisms underlying the effects of ADGRL2 on eNOS, it has to be noted that the binding of eNOS to Caveolin-1 inhibits its enzymatic activity and, thus, NO production, whereas interaction with Heat Shock Protein 90 (HSP90) enhances the activity of eNOS [22,23,24,25]. Therefore, we next investigated the role of ADGRL2 in eNOS binding to Caveolin-1 and HSP90 in ECs.

### 3.4. ADGRL2 Overexpression Reduces eNOS Interactions with Caveolin-1 and Enhances Binding to HSP90

**Figure 4:**
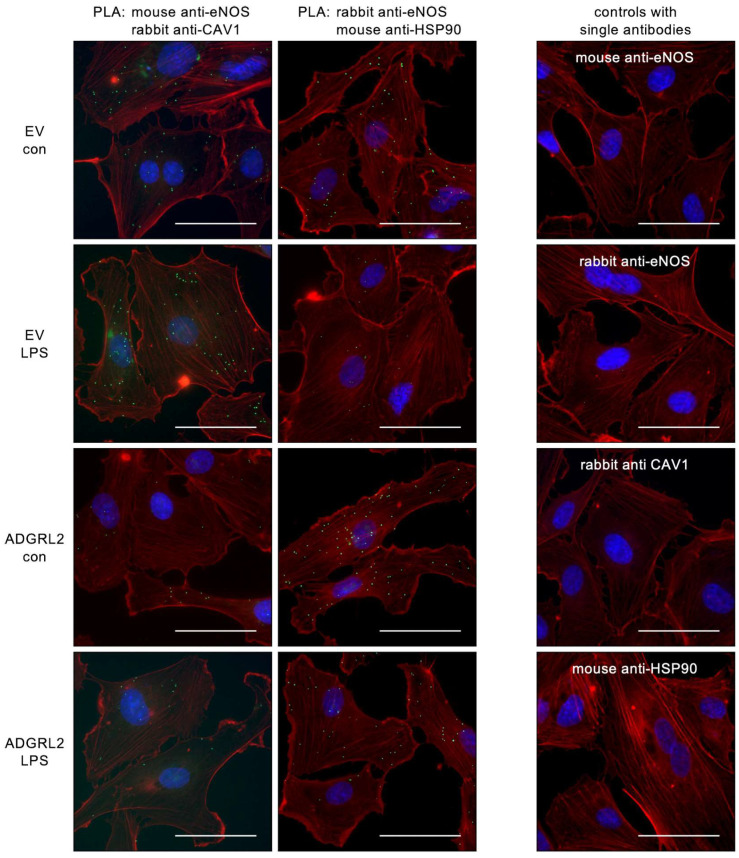
ADGRL2 alters eNOS interactions with Caveolin-1 and HSP90. ECs were transfected with an expression vector for *ADGRL2* or a corresponding empty vector (EV). After transfection, the cells were treated for 20 h with 150 ng/mL active LPS (LPS) or detoxified LPS as the control (con). Interactions between eNOS and Caveolin-1 (CAV1, left panels) and between eNOS and Heat Shock Protein 90 (HSP90, middle panels), respectively, were detected by a proximity ligation assay (green dots). The cytoskeleton was stained with Phalloidin Alexa 594 (red), and nuclei were counterstained with DAPI (blue). Negative controls, in which only one of the primary antibodies was used, are shown in the right panels (scale bars = 50 μm).

To determine potential changes in the interactions of eNOS with Caveolin-1 or HSP90, we performed proximity ligation assays (PLAs) in ECs transfected with EV or HA-ADGRL2-FLAG in the presence or absence of active LPS (Figure 4).

From the PLA data in Figure 4, it is obvious that LPS increased the binding of eNOS to Caveolin-1 and reduced its interactions with HSP90 in EV transfected cells. This is in perfect accordance with the previous observations regarding eNOS phosphorylation at S1177 and T495 (Figure 3), which indicated reduced activity. Interestingly, in cells overexpressing ADGRL2, the interaction between eNOS and Caveolin-1 was markedly diminished when compared to EV transfected cells. Moreover, ADGRL2 inhibited the LPS-induced increase in eNOS–Caveolin-1 interactions (Figure 4, left panels). Along the same lines, ADGRL2 maintained eNOS binding to HSP90 even after LPS treatment (Figure 4, middle panels), again corroborating the phosphorylation data (Figure 3). Thus, this aGPCR maintains the NO bioavailability even under LPS treatment by supporting the interaction of eNOS with HSP90, thereby keeping eNOS active.

In this context, it has to be noted that an increase in intracellular NO can lead to a dissociation of Kelch-like ECH-associated protein 1 (KEAP1) from NRF2 (NFE2-related factor 2), the master regulator of antioxidative defense. This leads to stabilization of NRF2 and nuclear translocation of this transcription factor. Active, nuclear NRF2, besides reducing oxidative stress, has been shown to inhibit EC activation and apoptosis [26], thereby contributing to proper EC functionality. Therefore, it is tempting to speculate that the improved eNOS activity induced by ADGRL2 could lead to increased NRF2 activity.

### 3.5. ADGRL2 Overexpression Enhances NRF2 Activity

**Figure 5:**
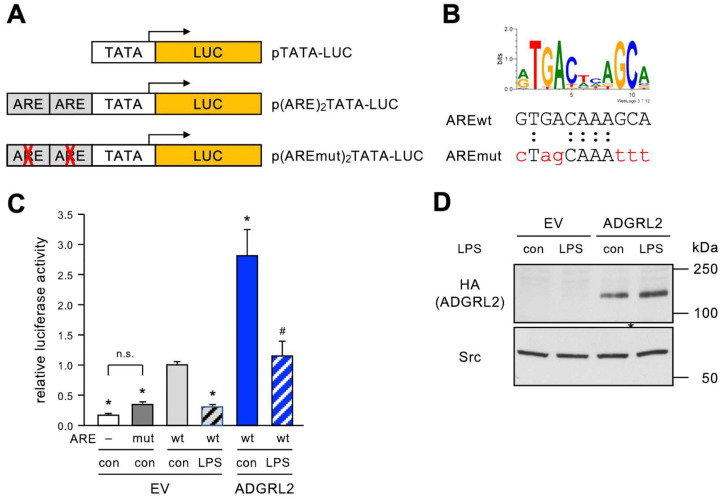
ADGRL2 activates endogenous NRF2 and protects against the LPS-induced loss of NRF2. (**A**) Schematic depiction of luciferase reporter vectors. In all plasmids, expression of firefly luciferase is driven by a minimal promoter derived from the herpes simplex virus thymidine kinase gene containing only a TATA box. The plasmid p(ARE)_2_TATA-LUC contains two copies of a 41 bl long antioxidant response element (ARE) from the murine Glutathione S-transferase alpha 1 gene promoter directly upstream of the minimal promoter. In p(AREmut)_2_TATA-LUC, the core sequence of these AREs is mutated to prevent the binding of NRF2. (**B**) Intact and mutated ARE. Shown is the sequence logo of the core binding sequence of human NRF2 and the corresponding sequence in the ARE of p(ARE)_2_TATA-LUC (AREwt); the mutated bases in p(AREmut)_2_TATA-LUC are shown in red (AREmut). (**C**,**D**) ECs were co-transfected with the reporter vector containing intact (wt), mutated (mut), or no AREs (–) and an expression vector for ADGRL2 or a or a corresponding empty vector (EV). After transfection, the cells were treated for 20 h with active LPS (LPS) or detoxified LPS as the control (con). (**C**) Luciferase activity was measured in cell lysates and is shown relative to AREwt/EV/con, which reflects the basal endogenous NRF2 activity (data are the mean ± SEM, n = 9–21, * *p* < 0.05 vs. AREwt/EV/con, # *p* < 0.05 vs. AREwt/EV/LPS, n.s. = not significant, one-way ANOVA with post hoc Tukey LSD test). (**D**) Expression of HA-tagged ADGRL2 (HA (ADGRL2)) in the co-transfections with p(ARE)_2_TATA-LUC was confirmed by immunoblot, and Src served as the loading control.

To investigate the effects of ADGRL2 on NRF2 activity, we first generated a NRF2-dependent luciferase reporter vector by inserting a tandem antioxidant response element (ARE), the binding site for NRF2, upstream of a minimal promoter containing only a TATA box driving expression of firefly luciferase. As a specificity control, we generated an identical vector in which bases within the AREs critical for NRF2 binding are mutated (Figure 5A,B).

These reporter plasmids were co-transfected with the HA-ADGRL2-FLAG expression vector or the corresponding empty vector, and the cells were treated with LPS as before. Then, luciferase activity as a direct readout for the transcriptional activity of NRF2 was measured (Figure 5C), and ADGRL2 overexpression was confirmed by immunoblot (Figure 5D).

The comparison of the luciferase reporter vector without ARE and the two vectors containing the intact and mutated AREs (Figure 5C, left three bars), respectively, demonstrated the specificity of the reporter system. While the construct with the intact AREs, which reflects basal endogenous NRF2 activity, yielded significantly higher luciferase activity than the one without AREs, the base mutations in the mutated ARE resulted in luciferase activity not different from the vector without AREs.

More importantly, LPS induced a complete loss of NRF2 activity in cells co-transfected with the empty vector instead of the *ADGRL2* expression vector. On the contrary, ADGRL2 overexpression had a striking effect on NRF2. Not only did it increase the basal NRF2 transcriptional activity nearly threefold but also protected the cells from complete loss upon LPS treatment, indicating that ADGRL2 can boost antioxidative defenses in ECs through the upregulation of NRF2 activity.

### 3.6. The Latrophilin Homolog LAT-2 Is Involved in the Control of Oxidative Stress in the Nematode C. elegans

**Figure 6:**
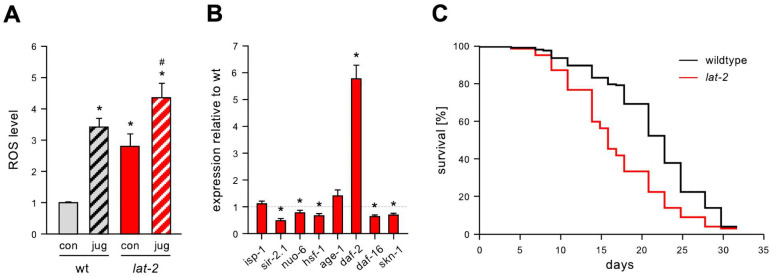
Nematodes lacking the latrophilin homolog *lat-2* show increased ROS levels, altered expression of stress-related genes, and reduced lifespans. (**A**) The ROS levels are increased in *lat-2(knu505)* individuals, both under normal conditions and after exposure to juglone. Worms were lysed after a 1-h treatment with 100 µM juglone (jug), incubated with H2DCF-DA, and DCF conversion was measured over a 2-h period. Control samples (con) were treated with ethanol instead of juglone (data are the mean ± SEM, n = 3, with 5 replicates per n, * *p* < 0.05 vs. wild-type/con, # *p* < 0.05 vs. wt/jug, two-way ANOVA with post hoc Bonferroni test for comparing multiple groups). (**B**) Expression of stress-related genes in *lat-2(knu505)* hermaphrodites. Expression of single genes was determined by semi-quantitative real-time PCR and normalized to the geometric mean of the reference genes *act*-*1*, *cdc*-42, *pmp*-3, *eif*-3.c, and *tba*-1 and is shown relative to wild-type animals (data are the mean ± SEM, n = 3, with 3 replicates per n, * *p* < 0.05 vs. wt, unpaired, two-sided Student’s *t*-test. (**C**) *lat-2(knu505)* nematodes have a significantly reduced lifespan (Cox–Mantel test, *p* < 0.0001). The median survival is 16 days for *lat-2(knu505)* hermaphrodites and 23 days for wild-type individuals (*p* < 0.001). n ≥ 200 in 4 independent experiments.

Latrophilins are evolutionarily highly conserved aGPCRs present in vertebrates and invertebrates, raising the question of whether the function in the protection against oxidative stress observed with mammalian ADGRL2 is conserved. In the nematode *C. elegans*, two homologs, *lat-1* and *lat-2*, exist, whereby no clear one-to-one homology between the mammalian and the nematode homologs can be determined. Previous work indicates though that, in terms of sequence, LAT-1 is most similar to ADGRL1 and also signals through the same G proteins [27]. Thus, we focused our analysis for functional similarities on LAT-2. Indeed, worm strain *lat-2(knu505)* (hereafter referred to as *lat-2* mutant) lacking the entire *lat-2* coding sequence and therefore, being a null mutant, had significantly higher levels of ROS than wild-type individuals (Figure 6A). This was the case for basal ROS amounts, as well as for those produced after stress induction using juglone, suggesting that, in the absence of the receptor, nematodes either produce more ROS or display a reduced ROS defense.

In order to gain further insights into this potential role of LAT-2 in oxidative stress defenses, we analyzed the expression of genes involved in stress protection or control of ROS: *nuo-6* and *isp-1* that code for mitochondrial proteins [28]; the transcription factors *hsf-1* [29], *skn-1* (homolog of NRF2), and *daf-16* (FOXO homolog) [30]; *daf-2*, which encodes a receptor upstream of DAF-16; and the aging-related genes *age-1* [31] and *sir-2.1* [32]. Most of these genes were differentially expressed in *lat-2* mutants (Figure 6B). Therefore, especially the ROS-protective gene *skn-1* [30] was significantly downregulated compared to the wild-type controls, which is consistent with the upregulation of NRF2 after overexpression of ADGRL2 observed in human ECs (Figure 5C).

Oxidative stress damage can severely affect the lifespan of *C. elegans* [30], and indeed, *lat-2* mutant nematodes display a significantly reduced lifespan (Figure 6C), further highlighting that LAT-2 has conserved protective functions across species. In order to exclude the possibility that the effect of *lat-2* deficiency on the lifespan is a secondary effect caused by, e.g., developmental defects, the brood size and survival to adulthood were determined. Both appeared to be indistinguishable from the wild-type individuals (Supplementary Figure S1).

## 4. Discussion

The major findings of the present study are the protective functions of ADGRL2 in endotoxemia-induced endothelial dysfunction, including increased eNOS activity and upregulation of antioxidative defense systems. The latter function is evolutionarily conserved, as *C elegans* lacking the latrophilin homolog LAT-2 have increased ROS levels and reduced the lifespan.

Our data demonstrate that, in primary human ECs, ADGRL2 preserves or even increases eNOS activity by shifting the binding of eNOS from Caveolin-1 to HSP90, resulting in decreased phosphorylation of T495 and the maintenance of phosphorylation of S1177 within eNOS. Interestingly, Human Cell Map (https://humancellmap.org (accessed on 13 October 2024)), a publicly available resource based on the characterization of the spatial organization of the proteome in HEK293 cells by BioID [33], lists an interaction between Caveolin-1 and ADGRL2. Thus, it is tempting to speculate that an increase in ADGRL2 in ECs sequesters Caveolin-1 by a direct interaction, making it unavailable for interactions with eNOS. In addition, there might be other mechanisms involved in eNOS activation through ADGRL2. It has been shown that Gαi2, one of the G proteins required for ADGRL2 signaling in ECs [7], is required for eNOS activation induced by dehydroepiandrosterone [34]. Taken these findings together, ADGRL2 seems to improve endothelial functionality not only by signaling via Gαi2 but also by changing the binding partners of eNOS.

The increased activation of eNOS induced by AGDRL2 results in protection against EC activation and apoptosis, as well as in maintenance of the migratory capacity. These diverse functions can be explained by an enhanced NO bioavailability induced by ADGRL2. It has been demonstrated that, in Caveolin-1-deficient mice, eNOS is more active, and the NO bioavailability is increased, resulting in the inhibition of LPS-induced ICAM1 expression [35]. This is in line with our data that ADGRL2 overexpression, on the one hand, leads to more active eNOS, most likely due to the reduced interaction between eNOS and Caveolin-1, and, on the other hand, completely inhibits LPS-induced ICAM1 upregulation and EC activation. With respect to apoptosis protection by ADGRL2, it has been demonstrated that S-nitrosation of Caspase-3 leads to its inactivation of ECs [36,37], resulting in a decreased apoptosis rate. Another important feature of ECs is their capacity to migrate, which is required when the monolayer is disrupted to preserve the endothelial barrier. Notably, the migratory capacity of ECs, another hallmark for their proper functionality, also depends on NO [38]. Taken together, these effects on the cellular level might be due to ADGRL2-mediated protection against the LPS-induced loss of eNOS activity.

Interestingly, not only Caspase-3 is S-nitrosated by NO in ECs. This modification of KEAP1 facilitates its dissociation from NRF2 and thereby contributes to the nuclear accumulation of NRF2 [26]. With our NRF2-dependent luciferase reporter system, we found that LPS treatment significantly reduced NRF2 activity in ECs. Moreover, in accordance with the positive effect of ADGRL2 on eNOS activity observed here, we also demonstrated that ADGRL2 significantly activates NRF2 in untreated ECs and completely restores NRF2 activity in LPS-treated cells. These data suggest that ADGRL2 leads not only to enhanced NO bioavailability but also to reduced oxidative stress due to NRF2 activation.

To address the question whether the protection against oxidative stress and the regulation of NRF2 by ADGRL2 exists across species, we investigated the role of the latrophilin homolog LAT-2 in the nematode *C. elegans* using a LAT-2-deficient strain. ROS levels in the *lat-2* mutant worms were already increased under normal maintenance, as well as after treatment with the ROS generator juglone. These data indicate that, in the absence of LAT-2, nematodes either produce more ROS or do not cope with the radicals well. In line with these results, expression of the genes involved in protection from oxidative stress is altered when LAT-2 is lacking. Thereby, most of the analyzed genes are downregulated, especially the ROS-protective gene *skn-1* [30], which is a homolog of mammalian NRF2. Although *C. elegans* does not have a vasculature, several mechanisms to protect from oxidative stress are conserved between mammals and the worm [39]. The function of SKN-1 is very similar to that of NRF proteins in response to oxidative stress, and *skn-1* is required for oxidative stress resistance and also longevity [40]. Its downregulation can indicate a reduced stress response in *lat-2* mutants, which could be one explanation for the increased ROS levels. This points towards a role of LAT-2 in the protection against oxidative damage like ADGRL2. Oxidative stress can result in a reduced lifespan [41], as is also the case for *lat-2* mutants. Thus, our *C. elegans* data are consistent with the positive impact of ADGRL2 on NRF2 in ECs.

It has to be noted that *C. elegans* harbors two latrophilin homologs, *lat-1* and *lat-2*. No information exists whether LAT-1 also has a function in the control of or protection against oxidative stress. Worms lacking LAT-1 display a plethora of phenotypes such as developmental arrest, only a very small number of individuals reach adulthood, and these have a reduced brood size and neurological defects [42,43]. Thus, delineating a potential role of LAT-1 in oxidative stress is challenging, as such effects, if present, might be secondary. This is in contrast to LAT-2, as the mutant worms seem not to have any developmental defects.

Future analyses will need to clarify whether LAT-2 in *C. elegans,* as well as ADGRL2 in primary human ECs, also have effects on aging and cellular senescence, respectively. As for *C. elegans*, the reduced lifespan of *lat-2* worms can be well explained by increased oxidative stress but might also have an age-related contribution. As for the primary ECs, the positive effects of ADGRL2 on eNOS and NRF2 activation points towards a protective role against cellular senescence induction.

## 5. Conclusions

In conclusion, ADGRL2 has protective effects across species. An increase in the ADGRL2 levels improves endothelial functionality, and this aGPCR could therefore be a promising therapeutic target for the treatment of sepsis to protect the endothelium and, thus, to restore endothelial cell integrity. Moreover, it seems to be of interest for the elderly population to determine whether potential protective functions of ADGRL2 also exist in aging and senescence.

## Data Availability

Raw data will be made available by the corresponding authors upon reasonable request.

## Supplementary Materials

The following supporting information can be downloaded at https://www.mdpi.com/article/10.3390/cells13221826/s1: Figure S1: *lat-2(knu505)* nematodes do not show any defects in brood size and survival to adulthood. Table S1: Antibodies used for immunoblots. Table S2: Antibodies used for immunostaining and PLA. Table S3: Primers used for semi-quantitative real-time PCR.
