# Supplementary material for: The Adhesion GPCR ADGRL2/LPHN2 Can Protect Against Cellular and Organismal Dysfunction
Source: Cells. 2024 Nov 5;13(22):1826. doi: 10.3390/cells13221826 (PMC11592504; doi:10.3390/cells13221826)
Supplement: Supplementary file 1 [file cells-13-01826-s001.zip › Suppl material.pdf]

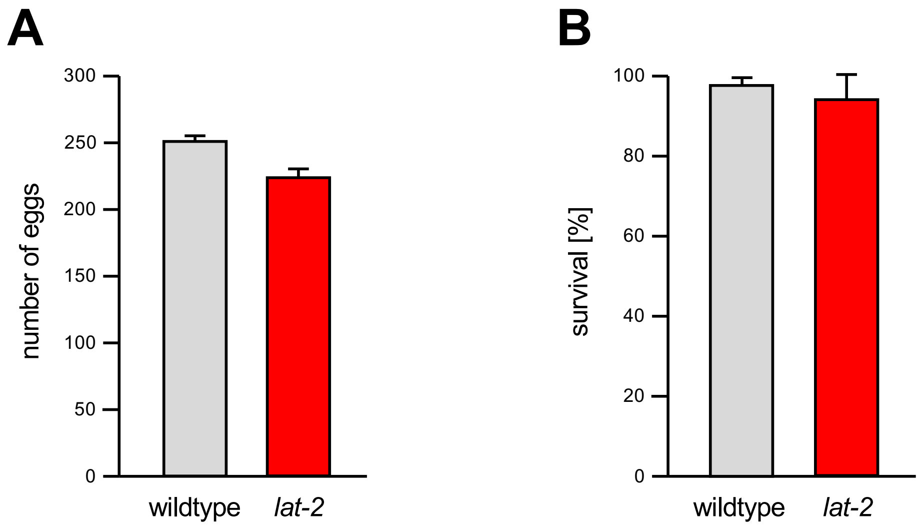

Supplementary figure 1: *lat-2(knu505)* nematodes do not show any defects in brood size and survival to adulthood. (**A**) Nematodes lacking LAT-2 function produce a brood size that is not significantly different to that of wild-type controls (data are mean ± SEM, n = 20 in 3 independent experiments, Student´s t-test). (**B**) Wild-type and *lat-2(knu505)* individuals develop to adulthood at similar, not significantly different rates (data are mean ± SEM, n = 20 in 3 independent experiments, unpaired, two-sided Student´s t-test).

Supplementary table 1: antibodies used for immunoblots.

primary antibodies

target protein	mono-/ polyclonal	species of origin	dilution	source	order nr.
Caspase-3	polyclonal	rabbit	1:1.000 (full-length Caspase-3)<br>1:300 (cleaved Caspase-3)	Cell Signaling Technology	9662
eNOS	monoclonal clone EPR19296	rabbit	1:2.000	Abcam	ab199956
Phospho-eNOS (Ser1177)	polyclonal	rabbit	1:500	Cell Signaling Technology	9571
Phospho-eNOS (Thr495)	polyclonal	rabbit	1:250	Cell Signaling Technology	9574
HA-tag	monoclonal clone GT4810	mouse	1:500	Sigma-Aldrich	SAB2702217
Src	monoclonal clone 32G6	rabbit	1:1.000	Cell Signaling Technology	2123

secondary antibodies

antibody	species of origin	dilution	source	order nr.
ECL anti-mouse IgG, horseradish peroxidase linked whole antibody	sheep	1:5.000	Cytiva	NA931
ECL anti-rabbit IgG, horseradish peroxidase linked whole antibody	sheep	1:5.000	Cytiva	NA934

Supplementary table 2: antibodies used for immunostaining and PLA

primary antibodies

target protein	mono-/ polyclonal	species of origin	dilution	source	order nr.
FLAG-tag	monoclonal clone M2	mouse	1:200	Sigma-Aldrich	F3165
FLAG-tag	monoclonal clone 8H8L17	mouse	1:200	Invitrogen	701629M
eNOS	monoclonal clone 6H2	mouse	1:100	Cell Signaling Technology	5880
eNOS	monoclonal clone EPR19296	rabbit	1:200	Abcam	ab199956
HSP90	monoclonal clone 68	mouse	1:250	BD Biosciences	610418
Caveolin-1	polyclonal	rabbit	1:200	Cell Signaling Technology	3238
ICAM-1	monoclonal clone 15.2	mouse	1:100	Santa Cruz Biotechnology	sc-107

secondary antibodies

antibody	species of origin	dilution	source	order nr.
anti-mouse IgG (H+L) Cross-Adsorbed Secondary Antibody, Alexa Fluor™ 488	goat	1:500	Invitrogen	A11001
anti-mouse IgG (H+L) Cross-Adsorbed Secondary Antibody, Alexa Fluor™ 594	goat	1:500	Invitrogen	A11005
anti-rabbit IgG (H+L) Cross-Adsorbed Secondary Antibody, Alexa Fluor™ 488	goat	1:500	Invitrogen	A11008
anti-rabbit IgG (H+L) Cross-Adsorbed Secondary Antibody, Alexa Fluor™ 594	goat	1:500	Invitrogen	A11012

Supplementary table 3: Primers used for semi-quantitative real-time PCR.
Shown are the sequences of the primers in 5'→3' direction and the expected amplification products.

transcript	primer		sequence		amplification product
human *ADGRL2*	hADGRL2 Ex17 for1	5'-	CTCTGGGCATCAAAGGAGCA	-3'	223 bp
	hADGRL2 Ex18/19 rev1	5'-	GGATCAATGTGTGGCAGGGT	-3'	
human *RPL32*	hmRPL32 Ex02 for1	5'-	GTGAAGCCCAAGATCGTCAA	-3'	257 bp
	hmRPL32 Ex03 rev1	5'-	TTGTTGCACATCAGCAGCAC	-3'	
C. elegans act-1	fwd_act-1	5'-	GCTGGACGTGATCTTACTGATTACC	-3'	114 bp
	rev_act-1	5'-	GTAGCAGAGCTTCTCCTTGATGTC	-3'	
C. elegans age-1	fwd_age-1	5'-	GACGGAACTCCCGACGTATC	-3'	567 bp
	rev_age-1	5'-	TCGTACGAGCCCAGAGAGAA	-3'	
C. elegans cdc-42	fwd_cdc-42	5'-	CTGCTGGACAGGAAGATTACG	-3'	111 bp
	rev_cdc-42	5'-	CTCGGACATTCTCGAATGAAG	-3'	
C. elegans daf-2	fwd_daf-2	5'-	GCTCTCGGAACAACCACTGA	-3'	132 bp
	rev_daf-2	5'-	GACAAGTCGAAGCCGTCTCA	-3'	
C. elegans daf-16	fwd_daf-16	5'-	CTAACTTCAAGCCAATGCCACTA	-3'	260 bp
	rev_daf-16	5'-	TCCAGCTTGACTCAGCTCATGTC	-3'	
C. elegans eif-3.c	fwd_eif-3.c	5'-	ACACTTGACGAGCCCACCGAC	-3'	191 bp
	rev_eif-3.c	5'-	TGCCGCTCGTTCCTTCCTGG	-3'	
C. elegans hsf-1	fwd_hsf-1	5'-	AAAATGGGGCAATTGGTGGC	-3'	333 bp
	rev_hsf-1	5'-	CACCTTGGGACAGTGGAGTC	-3'	
C. elegans isp-1	fwd_isp-1	5'-	AACGTCGTGCTCTTCCAACT	-3'	235 bp
	rev_isp-1	5'-	TTGGCAATCTCAGCCTTGGT	-3'	
C. elegans nuo-6	fwd_nuo-6	5'-	CTCCGAAACTGTGGCAGGAT	-3'	448 bp
	rev_nuo-6	5'-	GTTGGAGCAAAGAGACCGGA	-3'	
C. elegans pmp-3	fwd_ pmp-3	5'-	TGGCCGGATGATGGTGTCGC	-3'	190 bp
	rev_pmp-3	5'-	ACGAACAATGCCAAAGGCCAGC	-3'	
C. elegans sir-2.1	fwd_sir-2.1	5'-	GTCACGTGATAGTGGCAACG	-3'	544 bp
	rev_sir-2.1	5'-	AAGATCGGGAAACTCGCTCC	-3'	
C. elegans skn-1	fwd_skn-1	5'-	GACGTCAATTTATGGAGTGTCG	-3'	137 bp
	rev_skn-1	5'-	GAAGATGTTTTGTCGTGATCCG	-3'	
C.elegans tba-1	fwd_tba-1	5'-	GTACACTCCACTGATCTCTGCTGACAAG	-3'	149 bp
	rev_tba-1	5'-	CTCTGTACAAGAGGCAAACAGCCATG	-3'	
